# Transcriptome-based analysis of putative allergens of *Chorioptes texanus*

**DOI:** 10.1186/s13071-019-3843-7

**Published:** 2019-12-16

**Authors:** Ran He, Xiao-Bin Gu, Yue Xie, Xue-Rong Peng, Christiana Angel, Guang-You Yang

**Affiliations:** 1 Department of Parasitology, College of Veterinary Medicine, Sichuan Agricultural University, Chengdu, 611130 China; 2 Department of Chemistry, College of Life and Basic Science, Sichuan Agricultural University, Chengdu, 611130 China; 3 Department of Veterinary Parasitology, Faculty of Veterinary Sciences, Shaheed Benazir Bhutto University of Veterinary and Animal Sciences, Sindh, 67210 Pakistan

**Keywords:** Chorioptic mange, *Chorioptes texanus*, Transcriptome, RNA-Seq, Allergen, Hydrolase

## Abstract

**Background:**

Mites of the genus *Chorioptes* are non-burrowing and cause mange in a wide range of domestic and wild animals including cattle, horses, sheep, goats, panda, moose, camelids, mydaus and alpacas. Molecular biology and host-parasite interactions of *Chorioptes texanus* are poorly understood, and only a few *C. texanus* genes and transcript sequences are available in public databases including the allergen genes.

**Methods:**

*Chorioptes texanus* RNA was isolated from mites, and the transcriptome of *C. texanus* was analyzed using bioinformatics tools. *Chorioptes texanus* unigenes were compared with the allergen protein sequences from the mite allergen database website to predict the potential allergens. *Chorioptes texanus* putative allergen unigenes were compared with hydrolase genes by building a *C. texanus* hydrolase gene library with the best match of the homologous sequences. Three allergen genes were cloned and expressed, their recombinant proteins were purified and their allergenic activities were preliminarily investigated.

**Results:**

Transcriptome sequencing (RNA-Seq) of *C. texanus* was analyzed and results demonstrated that 33,138 unigenes were assembled with an average length of 751 bp. A total of 15,130 unigenes were annotated and 5598 unigenes were enriched in 262 KEGG signaling pathways. We obtained 209 putative allergen genes and 34 putative allergen-hydrolase genes. Three recombinant allergen proteins were observed to induce different degrees of allergic reactions on rabbit skin.

**Conclusions:**

The present transcriptome data provide a useful basis for understanding the host-parasite interaction and molecular biology of the *C. texanus* mite. The allergenic activities of recombinant *Euroglyphus maynei* 1-like (Eur m 1-like) protein, *Dermatophagoides ptreronyssinus* 1-like (Der p 1-like) protein and *Dermatophagoides ptreronyssinus* 7-like (Der p 7-like) protein were preliminarily investigated by intradermal skin test. Meanwhile, differences in eosinophil counts were observed in different injected sites of the skin. The identification of putative allergen genes and hydrolase genes offers opportunities for the development of new diagnostic, prevention and treatment methods.
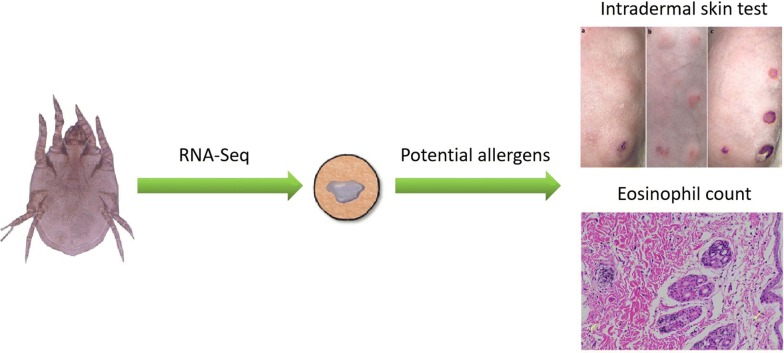

## Background

Parasitic mites belonging to the genus *Chorioptes* (Acariformes: Psoroptidae) are found worldwide, causing mange (chorioptic mange: a skin disease) in a wide range of domestic and wild animals [1, 2]. *Chorioptes* species are of considerable veterinary importance, as they commonly infest herbivorous animals, such as, cattle, horse, sheep, goat, moose, camelids, mydaus, alpacas and giant pandas [3–6]. *Chorioptes texanus* is a non-burrowing and obligatory mite species and passes its complete life-cycle on the same host. Although usually considered as a relatively less pathogenic type of mange, the pathology of chorioptic mange depends on duration and intensity of infestation and the susceptibility of the host [7]. It is usually localized to the legs, often infesting the base of the tail, perineum and the udder, where it is normally not easily observed [8, 9]. Chorioptic mange causes economic loses in both dairy and beef herds. The mites cause itching, rubbing and scratching which results in leather damage. Itching also makes the animal restless and can result in reduced production of beef and milk [10–12]. Based on morphological, epidemiological and genetic differentiation, *Chorioptes bovis* and *Chorioptes texanus* are generally accepted as two distinct valid species [1, 13, 14]. The egg, larva, protonymph, deutonymph and adult constitute a single life-cycle which spans approximately three weeks [7, 15]. Hosts may be asymptomatic at an early stage of infestation or low mite density, but mange may become generalized later or at a high mite density. Chorioptic mange is a highly seasonal disease, and more common in colder periods, particularly in winter and when cattle are stabled; however, it may recover when cattle return to pasture after the winter season [16]. Characteristic clinical signs include scratching and rubbing at the base of tail, perineum and legs [17]. The diagnosis of chorioptic mange is based on clinical signs and microscopic confirmation by identification of mites in the scrapings of infested skin from the host [18]. The control of chorioptic mange depends mainly on treatment with anti-parasitic drugs including eprinomectin, moxidectin, closantel, deltamethrin, ivermectin and selamectin [3, 10, 19]. Confirmatory laboratory diagnosis requires a longer time, and an overuse of the anti-parasitic drugs has led to environmental damage and drug resistance; therefore, it is necessary to find new efficient tools for diagnosis, prevention and treatment of this disease. *Chorioptes texanus* mites infest the host, eliciting an inflammation reaction [13], leading to an allergic skin response, hair loss, scratching and skin damage. Allergens are the main factors eliciting the hosts pro-inflammatory response, and most of the allergen genes are hydrolase genes, having crucial roles in mite evasion and survival in the host [20, 21]. Moreover, there are several allergen and hydrolase genes in mites which have been identified to be potential diagnostic antigens and vaccine candidates [22–25]. Intriguingly, analysis of *C. texanus* allergen and hydrolase genes may provide a valuable database for exploring new potential diagnostic, prevention and treatment methods. However, our current understanding of *C. texanus* molecular biology and host-parasite interactions is still incomplete. Additionally, available evidence regarding allergen genes of *C. texanus* are mainly from case studies and epidemiological and clinical reports, only a few *C. texanus* genes and transcript sequences are available in public databases [6, 26–28]. Therefore, in the present study, RNA-Seq was performed to analyze the transcriptome of *C. texanus* and to obtain a valuable sequence database of allergen and hydrolase genes. In addition, three allergen genes of *C. texanus* were subsequently cloned, expressed and their recombinant proteins were purified, and the allergenic activity was preliminarily investigated *via* an intradermal skin test.

## Methods

### Mite collection

*Chorioptes* spp. mites were isolated from scabs in the distal extremities and the base of the tail of three naturally infested cows from a farm in Chengdu, Sichuan Province, China. The animals were maintained under the same feeding and environmental conditions. Scabs were incubated in glass plates at 36 °C and mites were collected on an hourly basis. Mites that emerged from the scabs were collected and identified as *C. texanus* based on their morphology, as previously described [2, 26, 29] and were preserved immediately in liquid nitrogen (LN) after harvesting.

### RNA isolation and library construction

Total RNA was extracted from mite samples using Trizol reagent (Invitrogen, Carlsbad, USA) according to the manufacturer’s instructions. The purity and integrity of RNA was assessed by agarose gel electrophoresis and quantified by measuring the absorbance at 260/280 nm using a Nanodrop spectrophotometer (Bio-rad, California, USA). Sequencing libraries were obtained using the Illumina TruSeq^TM^ RNA sample preparation kits (Illumina, California, USA) following the manufacturer’s instructions and sequenced on an Illumina Hiseq 2000 platform (Illumina). Detailed methods are described in our previous study [30]. PCR products were purified using the AMPure XP system (Beckman Coulter, California, USA), and the quality of the sequencing libraries assessed on an Agilent Bioanalyzer 2100 (Agilent Technologies, California, USA).

### RNA-Seq data assembly and functional assignment

Raw reads were discarded if they contained more than 2-N bases, and further processed by stringent filtering steps by removing the adaptor sequences and low-quality reads. RNA-Seq data were assembled using Trinity (version: v2.8.0; parameter setting: min_kmer_cov is 2, other parameters are default parameters) (http://trinityrnaseq.sourceforge.net/) [31], and BLASTX alignment was carried out between unigenes and databases, including Nt (nucleotide sequences from NCBI), Nr (non-redundant protein sequences from NCBI), SwissProt (peer-reviewed protein sequence database), PFAM (protein family), GO (Gene Ontology) and KOG (eukaryote-specific version of the Clusters of Orthologous Groups). Coding sequences (CDS) were predicted by comparison with sequences from other eukaryotes. The CDS and direction of unigenes in databases were obtained based on the best alignment results. Unigenes which failed alignment to the databases were scanned with EST-Scan software to predict the CDS and direction [32]. The Blast2GO program and WEGO software were applied for further analysis [33, 34]. Blast2GO was employed to obtain GO annotations in the cellular component, molecular function and biological processes. Then WEGO was used to perform GO functional classification of unigenes. Unigene sequences were aligned to the above protein library according to the priority order of Nr and Swissprot. If the results matched, then the open reading frame (ORF) information of the transcript was extracted from the alignment results, and the coding region sequence was translated into an amino acid sequence according to the standard codon table (in the order of 5′–3′); otherwise the ORF of the unigenes was predicted by EST-scan software, thereby obtaining the nucleic acid sequence and amino acid sequence encoded by the part of a gene.

### Putative allergen genes and allergen-hydrolase genes

*Chorioptes texanus* unigenes were compared with the allergen protein sequences from the allergen database website (http://www.allergome.org), and putative allergen gene sequences were obtained. Additionally, *C. texanus* putative mite allergen unigenes were compared with the hydrolase gene sequences by building a *C. texanus* hydrolase gene library with the best match of the homologous sequences.

### Cloning and expression of three allergen genes and purification of their recombinant protein

The putative allergen genes were obtained after conducting a BLAST search, and three allergen genes, i.e. *Dermatophagoides ptreronyssinus* 1-like (Der p 1-like) protein gene, *Dermatophagoides ptreronyssinus* 7-like (Der p 7-like) protein gene and *Euroglyphus maynei* 1-like (Eur m 1-like) protein gene, were selected. The cDNA was transcribed using a RevertAi^TM^ First Strand cDNA Synthesis Kit (Thermo, Massachusetts, USA) according to the manufacturer’s instructions and stored at − 80 °C. Available gene sequences were used to design primers for three genes as follows, Der p 1-like protein gene was amplified from cDNA using a sense primer (5′-CGC GGA TCC AAA AAA ATG AAA ATC ATA TCA TCA ATC GCA ATT TTT TCG CT-3′) containing a *BamH*I site (underlined) and antisense primer (5′-CCG CTC GAG TCA AAC AGA TAA AAC AAC ATA TGG ATA TTG TTC AAT GCC-3′) containing a *Xho*I site (underlined); Der p 7-like protein gene was amplified from cDNA using a sense primer (5′-CGC GGA TCC GAT CCA ATT CAC TTT GAT AAA AT-3′) containing a *BamH*I site (underlined) and antisense primer (5′-CCG CTC GAG TAA TGT TGA TCG ATT GCT TTT TT-3′) containing a *Xho*I site (underlined); Eur m 1-like protein gene was amplified from cDNA using a sense primer (5′-CGC GGA TCC CGT CCA TCA TCA ATT AAA AC-3′) containing a *BamH*I site (underlined) and antisense primer (5′-CCG CTC GAG TTA AAG AAT AAC AAC ATA TGG ATA T-3′) containing a *Xho*I site (underlined). The cDNA encoding the Der p 1-like protein gene and Der p 7-like protein gene were successfully sub-cloned into the pET32a(+) expression vector (Novagen, Darmstadt, Germany), and the Eur m 1-like protein gene was successfully sub-cloned into the pET28a(+) expression vector and expressed in *Escherichia coli* BL21(DE3). The expressed recombinant proteins were purified using Ni^2+^ affinity chromatography (Bio-Rad, California, USA) according the manufacturer’s instructions.

### Intradermal skin test and eosinophil count

An intradermal skin test was performed to investigate the allergenic activity of recombinant allergen proteins. Nine New Zealand White rabbits were injected subcutaneously with 100, 200, 400 μg of purified recombinant allergen protein in 0.1 ml PBS. The histamine-injected group (4 mg/ml, 0.1 ml) was used as a positive control. The negative controls were: (i) 0.1 ml PBS; (ii) physiological saline (0.9%, 0.1 ml); and (iii) 400 μg of purified pET-32a (+) empty expression vector in 0.1 ml PBS. Skin reactions were recorded every 30 min following an injection, and when the presence of a wheal and erythema was observed the skin reactions were defined as positive. After 2.5 h, skin samples were collected with an annular skin sampler (diameter of 7 mm), and fixed in 4% paraformaldehyde in PBS (pH 7.4) for 24 h. A rotary microtome was used to serially cut the paraffin-embedded specimens at 5 μm thickness and sections were mounted on clean glass slides. H&E staining was performed to observe differential eosinophilic infiltration at different injected sites of the skin. For each slide, six microscopic fields (200×) were randomly selected and microphotographed. Image-Pro Plus 6.0 was used to count the number of eosinophils per field.

### Validation of RNA-Seq data by RT-qPCR

RT-qPCR analysis was performed to validate the RNA-Seq results. Part of allergen genes were validated according to the comparison between *C. texanus* putative allergens genes and genes from the allergen database website. A MX300P spectrofluorometric thermal cycler (Stratagene, California, USA) was used to conduct the RT-qPCR. The cycling conditions were as follows, an initial denaturation at 95 °C for 2 min, followed by 40 cycles at 94 °C for 20 s and 58 °C for 20 s. qPCR was conducted in triplicate and the relative gene expression levels were calculated using the 2^−ΔΔCq^ method [35].

## Results

### RNA-Seq and assembly of *C. texanus* transcriptome data

RNA-Seq analysis of *C. texanus* yielded 48,497,838 raw reads from mites (GenBank project accession no. PRJNA495065). After quality assessment and filtering, 46,404,816 clean reads were obtained with a Q20 of 96.66% and a GG of 33.48%. Clean reads were used for assembly with Trinity software. A total of 51,868 transcripts with N50 values of 2406 and 33,138 unigenes with N50 values of 1340 were generated respectively (Additional file 1: Figure S1).

### Functional annotation and GO classification

Unigenes were annotated with multiple databases using different software and/or websites. After final assembly, 33,138 unigenes were compared to NR, NT, SwissProt and KOG using NCBI blast 2.2.28+ with an E-value cut-off of le-5 for NR, NT, SwissProt and E-value, cut-off of le-3 for KOG. There were 7998 unigenes categorized into 25 molecular families when aligned through the KOG database, and unigenes distribution is shown in Additional file 2: Figure S2. Furthermore, unigenes were analyzed using the HMMER 3.0 package and hmmscan with the PFAM database, and were analyzed within Blast2GO with the GO database and annotated by KAAS, KEGG Automatic Annotation Server with the KEGG database (Table 1). A total of 11,915 unigenes were mapped to GO terms which were categorized into biological processes (BP), molecular functions (MF) and cellular components (CC) (Additional file 3: Figure S3). The top three predominant terms for BP were cellular process, metabolic process and single-organism process; whereas the top three terms in MF were binding, catalytic activity and transporter activity; and for CC, the top three terms were cell, cell part and organelle.

**Table 1:** Summary of assembled unigenes annotation

Annotation method	No. of unigenes	Percentage (%)
Annotated in NR	10,508	31.7
Annotated in NT	4113	12.41
Annotated in KO	5598	16.89
Annotated in SwissProt	9365	28.26
Annotated in PFAM	11,309	34.12
Annotated in GO	11,915	35.95
Annotated in KOG	7148	21.57
Annotated in all databases	1495	4.51
Annotated in at least one database	15,130	45.65
Total unigenes	33,138	100

### KEGG pathway analysis of unigenes

KEGG pathway enrichment analysis revealed that a total of 5598 unigenes were enriched in 262 pathways. The KEGG pathways included five categories, cellular processing (*n* = 17; 6.5%), environmental information processing (*n* = 31; 11.8%), genetic information processing (*n* = 22; 8.4%), metabolism (*n* = 123; 46.9%) and organismal systems (*n* = 69; 26.3%) (Additional file 4: Figure S4). Transport and catabolism (443 unigenes) was the most abundant subcategory in cellular processing; signal transduction (1393 unigenes) was the most abundant subcategory in environment information processing; translation (681 unigenes) was the most abundant subcategory in genetic information processing; carbohydrate metabolism (558 unigenes) was the most abundant subcategory in metabolism and organismal systems; endocrine system (947 unigenes) was the most abundant subcategory in organismal systems. The top 10 unigenes abundant pathways were ribosome (381 unigenes), carbon metabolism (182 unigenes), protein processing in endoplasmic reticulum (181 unigenes), oxidative phosphorylation (161 unigenes), PI3K-Akt signaling pathway (146 unigenes), biosynthesis of amino acids (145 unigenes), spliceosome (143 unigenes), MAPK signaling pathway (137 unigenes), purine metabolism (136 unigenes), and phagosome (130 unigenes). CDS were determined using the BLASTX program and EST-Scan program, unigenes length and unigenes counts are shown in Additional file: 5 Figure S5 and Additional file 6: Figure S6, respectively.

### Putative allergen genes and allergen-hydrolase genes

According to results from the allergen blast, 1246 allergen homologous unigenes were obtained from the *C. texanus* RNA-Seq data. The number of unigenes homologous to allergens from the website was 1167. From these, 959 unigenes were non-mite putative allergens and 208 unigenes were putative allergens of seven species of mite, i.e. *Dermatophagoides farina*, *Dermatophagoides pteronyssinus*, *Euroglyphus maynei*, *Lepidoglyphus destructor*, *Psoroptes ovis*, *Sarcoptes scabiei*, *Tyrophagus putrescentiae* and *Acarus siro*. A hydrolase gene sequence bank was constructed using the homologous hydrolase sequences from the *C. texanus* RNA-Seq data and 34 of them were putative allergen genes of *C. texanus*, including comp7640_c0, comp2015_c0, comp11218_c1, comp14904_c0, comp14553_c12, comp4959_c0, etc. The top 15 putative allergen genes were listed according to the fragments per kilobase of exon per million parts mapping (FPKM) value (Table 2) [36].

**Table 2:** Putative allergen unigenes

Unigene ID	Allergen protein ID	Species	Description	FPKM
comp14834_c0	Q66RP5	*Tyrophagus putrescentiae*	Q66RP5_TYRPU Fatty acid-binding protein	4567.35
comp8879_c0	Q965E2	*Psoroptes ovis*	ALL2_PSOOV Mite group 2 allergen Pso o 2	4012.6
comp5529_c0	Q3BJY7	*Psoroptes ovis*	Q3BJY7_PSOOV Putative tropomyosin	2488.73
comp14847_c0	Q58A71	*Dermatophagoides farinae*	Q58A71_DERFA Der f 1 allergen preproenzyme	2403.79
comp14391_c0	B2ZSY4	*Dermatophagoides pteronyssinus*	B2ZSY4_DERPT Der p 20 allergen	2048.65
comp14418_c3	A0A088SAS1	*Dermatophagoides farinae*	A0A088SAS1_DERFA Der f 28 allergen	1676.1
comp14857_c1	E0A8N8	*Dermatophagoides pteronyssinus*	E0A8N8_DERPT Der p 13 allergen	1408.55
comp14868_c0	A0A088SAG5	*Dermatophagoides farinae*	A0A088SAG5_DERFA Der f 30 allergen	1269.13
comp14714_c1	Q52PV9	*Tyrophagus putrescentiae*	Q52PV9_TYRPU Alpha-tubulin	1123.25
comp14871_c0	L7N6F8	*Dermatophagoides pteronyssinus*	L7N6F8_DERPT Dust mite allergen	1042.08
comp14617_c13	Q8WQ47	*Lepidoglyphus destructor*	TBA_LEPDS Tubulin alpha chain	995.42
comp9497_c1	Q6Y2F9	*Dermatophagoides pteronyssinus*	Q6Y2F9_DERPT HDM allergen	983.99
comp14890_c0	A1KXH1	*Dermatophagoides farinae*	A1KXH1_DERFA Der f 13 allergen	953.76
comp14877_c0	Q2L7C5	*Dermatophagoides pteronyssinus*	Q2L7C5_DERPT Allergen	934.56
comp14623_c9	Q9U785	*Euroglyphus maynei*	Q9U785_EURMA High molecular weight allergen M-177	904.88

### Cloning and expression of three allergen genes and purification of their recombinant proteins

The cDNA encoding Der p 1-like protein, Der p 7-like protein and Eur m 1-like protein contained 984-bp, 660-bp and 915-bp ORF, respectively. The cDNA encoding these three allergen genes were successfully sub-cloned into expression vectors and expressed in *E. coli* BL21 (DE3) cells with a molecular weight of approximately 56 kDa (Der p 1-like protein; including the His tag), 44 kDa (Der p 7-like protein; including the His tag) and 42 kDa (Eur m 1-like protein; including the His tag). Then recombinant proteins were purified using a Ni-chelating column and examined by SDS-PAGE.

### Intradermal skin test and eosinophil count

**Fig. 1:**
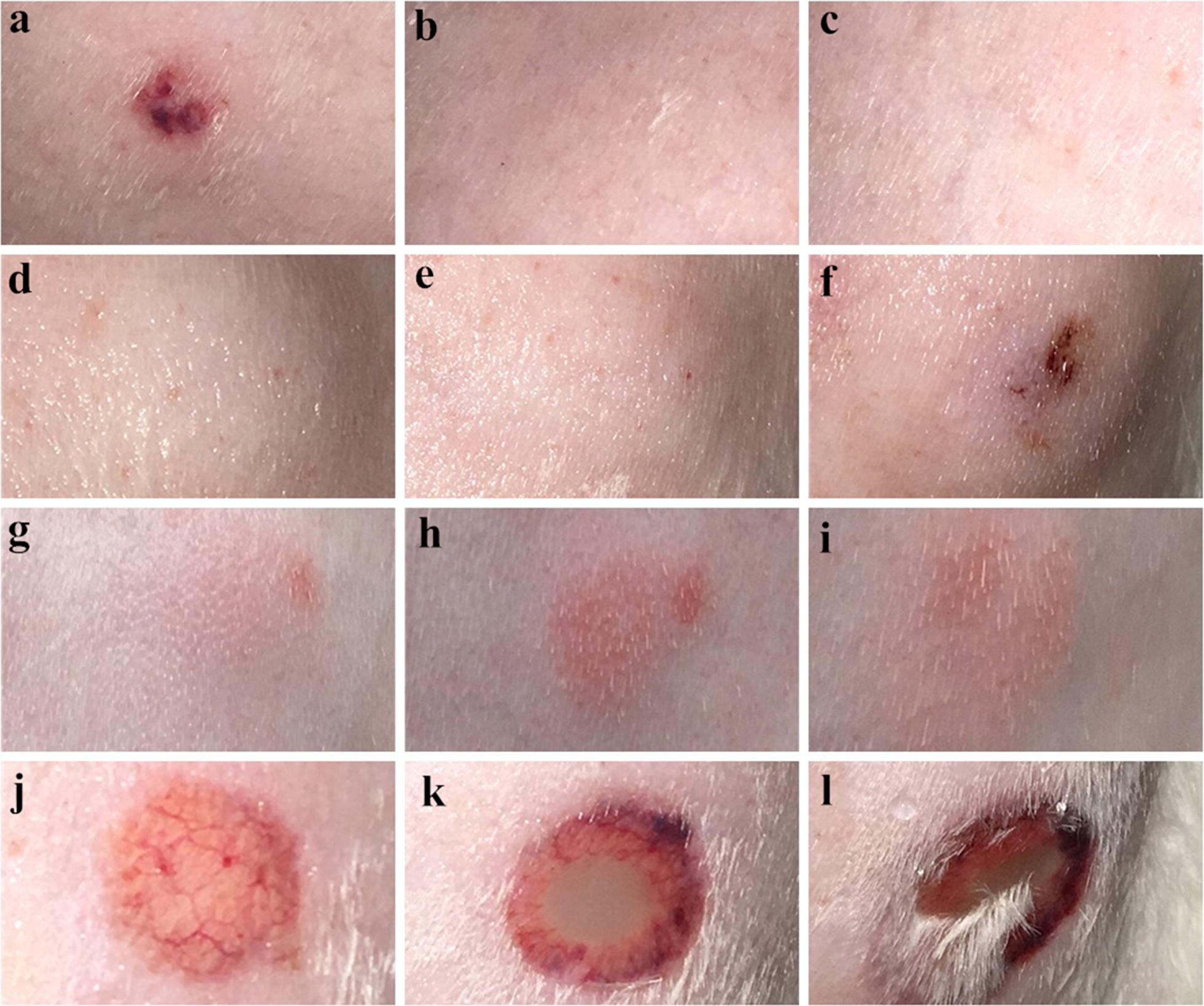
Intradermal skin test. **a** Histamine (4 mg/ml, 0.1 ml). **b** 400 μg of purified pET-32a (+) empty expression vector in 0.1 ml PBS. **c** 0.1 ml PBS. **d** 100 μg of purified recombinant Der p 1-like protein. **e** 200 μg of purified recombinant Der p 1-like protein. **f** 400 μg of purified recombinant Der p 1-like protein. **g** 100 μg of purified recombinant Der p 7-like protein. **h** 200 μg of purified recombinant Der p 7-like protein. **i** 400 μg of purified recombinant Der p 7-like protein. **j** 100 μg of purified recombinant Eur m 1-like protein. **k** 200 μg of purified recombinant Eur m 1-like protein. **l** 400 μg of purified recombinant Eur m 1-like protein

**Fig. 2:**
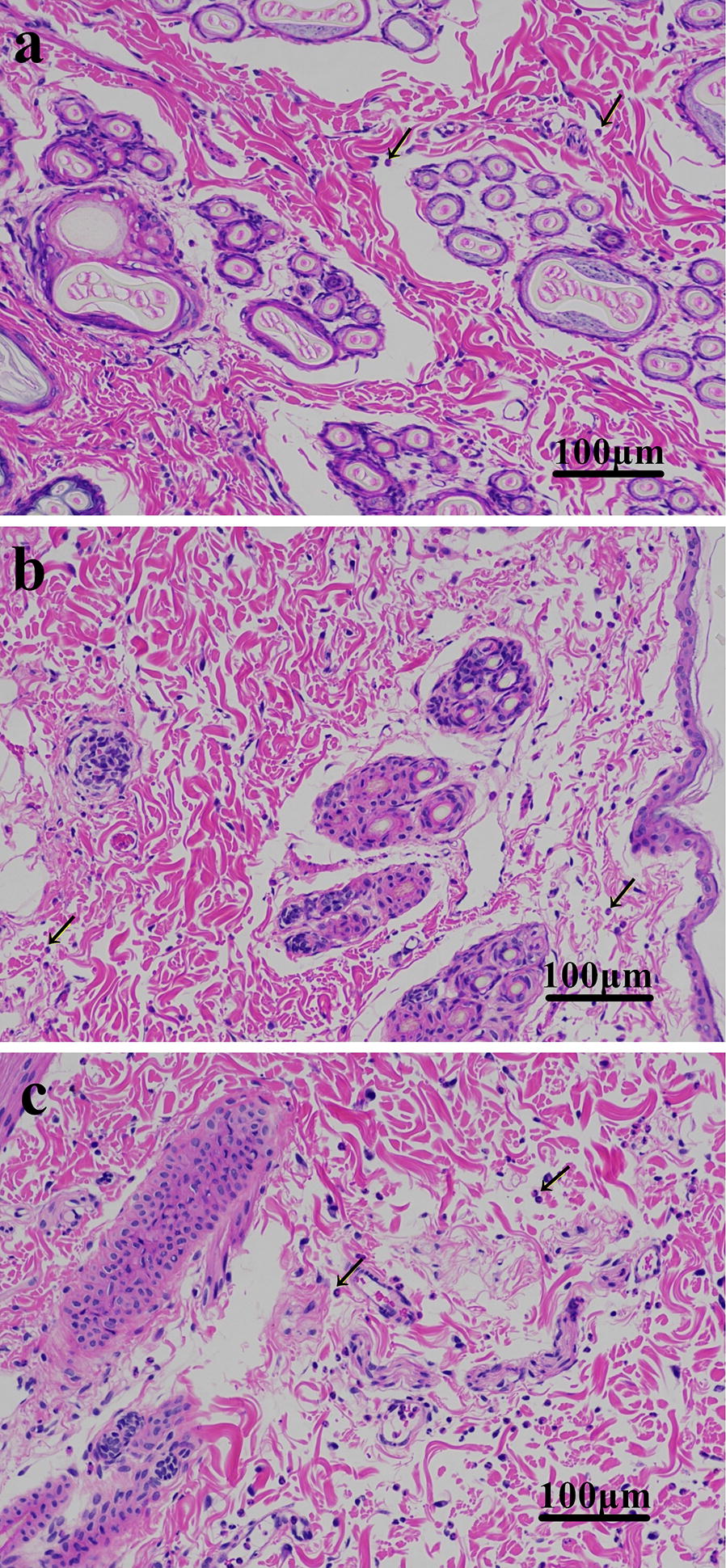
Hematoxylin and eosin (H&E) staining. **a** H&E staining of skin injected with recombinant Der p 1-like protein. **b** H&E staining of skin injected with recombinant Der p 7-like protein. **c** H&E staining of skin injected with recombinant Eur m 1-like protein. Arrows indicate eosinophils

Recombinant Eur m 1-like protein injected at 100 μg, 200 μg and 400 μg doses produced a wheal, flare and erythema reaction, including a blistering and ulceration reaction. As for the recombinant Der p 1-like protein, 400 μg recombinant protein produced erythema reaction, whereas recombinant Der p 7-like protein at 100 μg, 200 μg and 400 μg doses produced a wheal and erythema reaction. No reaction was induced by 0.9% physiological saline, PBS or empty expression vector, whereas the histamine-positive control produced a wheal reaction (Fig. 1). Histological assessment revealed an increased infiltration of eosinophils at the site of recombinant protein injection compared to the 0.9% physiological saline-injected site. As for the different recombinant protein-injected sites, the number of eosinophils in the Eur m 1-like protein site was greater compared to the Der p 1-like protein and Der p 7-like protein-injected sites, whereas eosinophil counts in Der p 1-like protein site showed similarity to that in the Der p 7-like protein-injected sites (Fig. 2).

### RT-qPCR validation

**Fig. 3:**
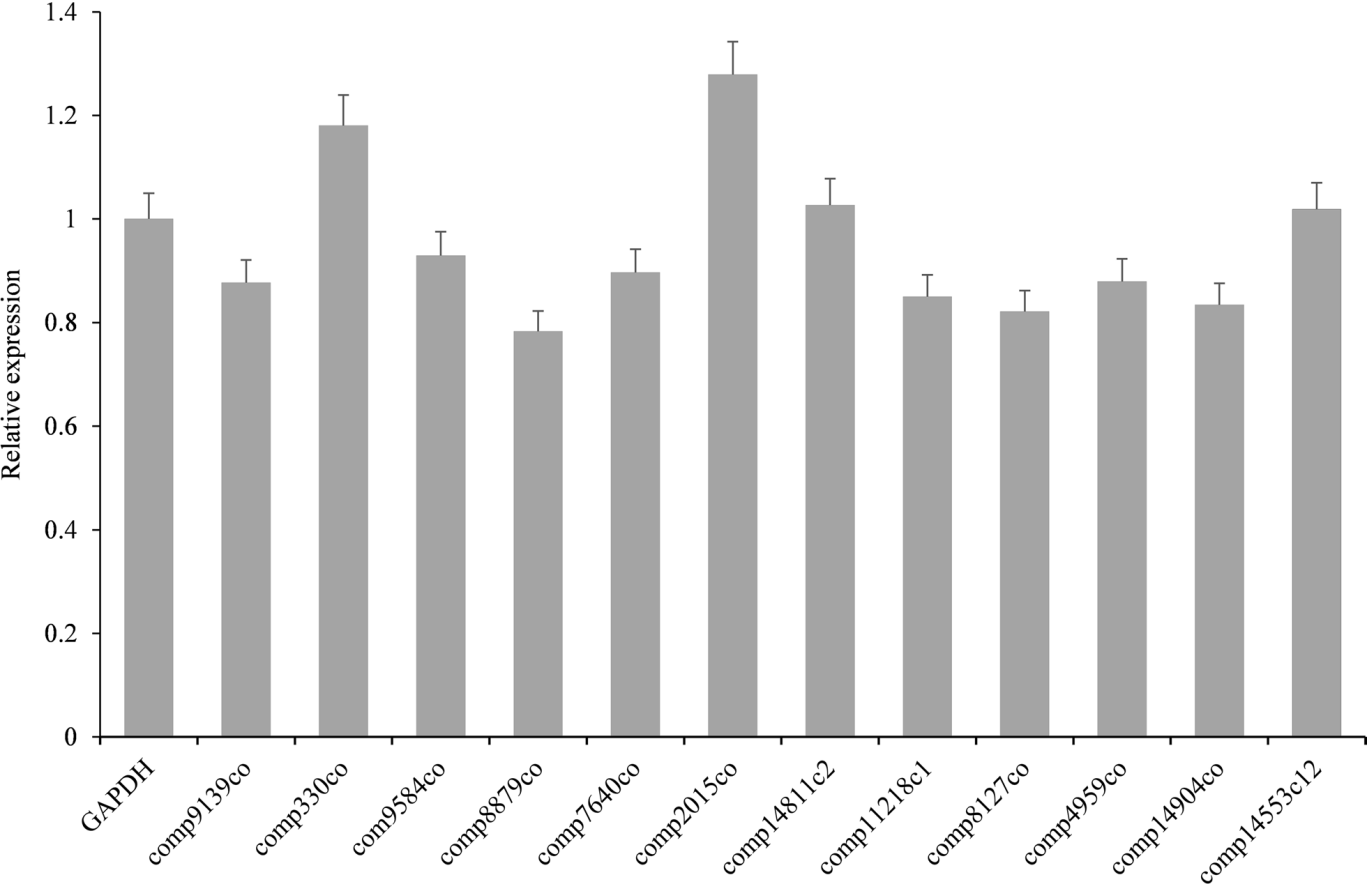
RT-qPCR validation of selected *C. texanus* unigenes. qPCR was conducted in triplicate

RT-qPCR was performed to validate allergen unigenes, which showed that our results were highly correlated with transcripts obtained by RNA-Seq, revealing that our RNA-seq results were reliable. Primers of allergen unigenes and the internal reference gene GAPDH were designed for RT-qPCR. Expression of 12 allergens (comp7640_c0, comp2015_c0, comp14904_c0, comp14553_c12, comp4959_c0, comp8127_c0, comp9584_c0, comp330_c0, comp8879_c0, comp11218_c1, comp9139_c0, and comp14811_c2) were normalized to GAPDH (Fig. 3).

## Discussion

Unigenes of *C. texanus* were enriched in 262 signaling pathways as shown in KEGG pathway enrichment analysis. The enriched pathways including immune related signaling pathways, MAPK signaling pathway, chemokine signaling pathway (90 unigenes), Fc gamma R-mediated phagocytosis (61 unigenes), Toll-like receptor signaling pathway (51 unigenes), NOD-like receptor signaling pathway (46 unigenes), Jak-STAT signaling pathway (34 unigenes), RIG-I-like receptor signaling pathway (23 unigenes) and others. From these, the Jak-STAT signaling pathway, Toll-like receptor signaling pathway, and immune deficiency signaling pathway are reported to be the main signaling pathways of the insect innate immune response [37, 38]; however, the immune deficiency signaling pathway was not identified in the KEGG pathway analysis result. Jak-STAT controls multiple biological processes in metazoan development and tissue homoeostasis, and it has been associated with several aspects of the innate immune system [39]. The Jak-STAT signaling pathway can affect the activation of neutrophils and macrophages, pro-inflammation response, and regulate B cell and T cell differentiation [40, 41]. Toll-like receptor signaling has been studied by various approaches involving genetic, biochemical, structural, cell biology and bioinformatics studies [42, 43]. Toll-like receptors (TLRs), are an important family of pattern recognition receptors (PRRs) and are responsible for the recognition of pathogen-associated molecular patterns from infectious pathogens. PRRs activate downstream signaling pathways which lead to the induction of innate immune responses by producing the inflammatory cytokines, type I interferon (IFN), and other mediators [44]. These processes not only trigger immediate host defensive responses such as inflammation, but also prime and orchestrate the antigen-specific adaptive immune responses [44]. Toll-like receptor signaling appears to be divergent and plays an important role in many aspects of the innate immune responses in many pathogens [43, 45, 46].

*Chorioptes texanus* infested cattle suffer hair loss, scratching and skin damage. The pro-inflammatory cytokines commonly cause skin damage and these factors come from the secretions/excretions of mites. Secretions from some parasites are important allergens which elicit a pro-inflammatory response in the host [47–49]. Although allergic reactions in host can lead to a protective immune response, allergic events can lead to immunological hypersensitivity, tissue damage and may produce harmful effects. IgE antibody mediates a type I immunological hypersensitivity which is commonly caused by allergenicity of an allergen [50, 51]. The ability of an antigen to induce allergic sensitization is called allergenicity, which is measured by the reactivity of allergen-induced IgE antibody, and indicates that immune system of the host has been elicited to an allergic state [52–54]. Twenty-one allergens have been widely studied (structural, chemical and biological properties) in house dust mites [54, 55]. Allergens can activate the innate immune cells and induce immunologic responses by binding to C-type lectin receptors or Toll-like receptors [56]. In addition, group 1 and 2 allergens from *Dermatophagoides pteronyssinus* were reported to boost the innate immune response and cleave IgE receptors, resulting in an increased allergic reaction in the host [57–59]. Several other allergens have been predicted in the transcriptome or genome of *S. scabiei* and *P. ovis*, including triosephosphate isomerase, chitinase-like protein of *S. scabiei* and Pso 1, Pso 2, Pso 10, Pso 11 of *P. ovis* [23, 60–63]. These findings highlight that mite allergens may also play a crucial role in the pathogenesis of chorioptic mange. Proteases are mainly allergens of the house dust mite and hydrolysis of these proteases is reported to promote and aggravate the allergic reaction and inflammatory responses [20, 21, 64, 65]. Proteases can degrade fibrinogen in *P. ovis*, and provide flow of serous exudate from the host during mite feeding [21]. Additionally, aspartic protease of *S. scabiei* can digest serum molecules and skin of the host [66]. Similarly, hydrolases may also contribute to mite survival and invasion, and have a crucial role in the interaction of *C. texanus* mites and their host. In the present study, comparisons between *C. texanus* unigenes and the allergen website resulted in 209 putative mite allergen unigenes homologous to the allergen website. Also 34 putative allergen-hydrolase unigenes were obtained. Normally IgE ELISA and IgE dot blotting are performed to identify the allergenic activity of allergens. However, in the present study, IgE ELISA and IgE dot blotting were not feasible due to the lack of an effective secondary anti-cow IgE antibody. Three allergen genes were selected from the 209 putative mite allergen unigenes, Der p 1-like protein gene, Der p 7-like protein gene and Eur m 1-like protein gene. Der p 1 and group 1 allergens from *Dermatophagoides farina* represent a homologous pair of major allergens which possess both cross-reacting and species-specific epitopes [67], group 1 allergen (Der p 1) from *Dermatophagoides pteronyssinus* has been reported to boost the innate immune response and cleave IgE receptors which may have increased the allergic reaction in the host [57–59]. Recombinant fusion protein of Der p 1 activates basophils in mite-allergic patients and triggers specific CD4+ T cell proliferation [68]. Interestingly, although Eur m 1 showed 85% amino acid identity with Der p 1 [69, 70], but 100 μg, 200 μg and 400 μg of recombinant Eur m 1-like protein produced the wheal, flare and erythema reaction, even blistering and ulceration, and only 400 μg recombinant Der p 1-like protein produced an erythema reaction. There is a large degree of T cell cross-reactivity between the whole purified allergen from each species, according to the proliferative and cytokine response to the group 1 and group 7 allergens [71]. Der p 7 is a glycoprotein and performs its function partially through glycan binding, it can activate BMDCs through TLR4 and DC-SIGN, and establish a link between innate TLR4-C-type lectin receptors and adaptive Th2 immunity [72]. Normally, the frequency of IgE-binding to the allergen in sera from an allergic population is used to determine the relative importance of the individual house dust mite allergens, and the equivalent increased IL-5 response of PBMC to group 7 and group 1 allergen (different IgE-inducing activity) indicates that allergens may be equally capable of contributing to an asthmatic response by inducing eosinophilia [73]. Der p 7 has a high IgE-binding activity but only reacts with 50% allergic sera, and this may explain the onset of a mild allergic skin reaction compared to the Eur m 1-like protein. Eosinophils participate in the adaptive immune response as antigen presenting cells and secrete Th cell chemokine, accounting about 1–3% in normal physiological conditions. Normally eosinophils are not present in skin, but many contributing factors, including hypersensitivity to arthropod bites and parasites, can cause eosinophilic infiltration in the skin, resulting in skin disorders. In the present study, we observed that the number eosinophils in the Eur m 1-like protein-injected sites was higher than in the Der p 1-like protein and Der p 7-like protein-injected sites. These findings indicate that, these recombinant proteins can induce allergic reactions, further validating the reliability of our sequencing results and analysis. The Der p 1-like protein was a low-range allergen, the Der p 7-like protein and Eur m 1-like proteins were medium-range and high-range allergens in *C. texanus* mite, respectively. Further focused functional studies on these genes can improve our understating of *C. texanus* and host interactions, which may contribute to the discovery of novel interventions against this ecoparasite.

## Conclusions

Comparisons between *C. texanus* unigenes and the allergen database website resulted in 209 putative mite allergen unigenes. Also 34 putative hydrolase-allergen unigenes were obtained. The allergenic activity of recombinant Eur m 1-like protein, Der p 1-like protein and Der p 7-like protein were preliminarily investigated by an intradermal skin test. The transcriptome of *C. texanus* provides a useful basis for understanding the host-parasite interaction and molecular biology of mites. Identification of putative allergen genes and hydrolase genes could offer opportunities for the development of new efficient diagnostic, prevention and treatment methods.

## Supplementary information

**Additional file 1: Figure S1.** Length distribution of *C. texanus* unigenes and transcript.

**Additional file 2: Figure S2.** KOG classification of *C. texanus* unigenes.

**Additional file 3: Figure S3.** GO annotation of *C. texanus* unigenes.

**Additional file 4: Figure S4.** KEGG pathway analysis of *C. texanus* unigenes.

**Additional file 5: Figure S5.** Length distribution of CDS determined by Blastx program.

**Additional file 6: Figure S6.** Length distribution of CDS determined by EST-Scan software.

## Data Availability

The transcriptome raw sequence data has been submitted to the GenBank (Project Accession No. PRJNA495065). The other data supporting our findings and conclusions are available in the article and its additional files.

## Abbreviations

GO: Gene Ontology
KEGG: Kyoto Encyclopedia of Genes and Genomes
KO: KEGG Orthology
KOG: Eukaryotic Orthology Groups Database. CDS: coding sequences
RT-qPCR: reverse-transcription quantitative PCR
