# Supplementary figures and images for: Transcriptome-based analysis of putative allergens of Chorioptes texanus
Source: Parasit Vectors. 2019 Dec 16;12:587. doi: 10.1186/s13071-019-3843-7 (PMC6916059; doi:10.1186/s13071-019-3843-7)

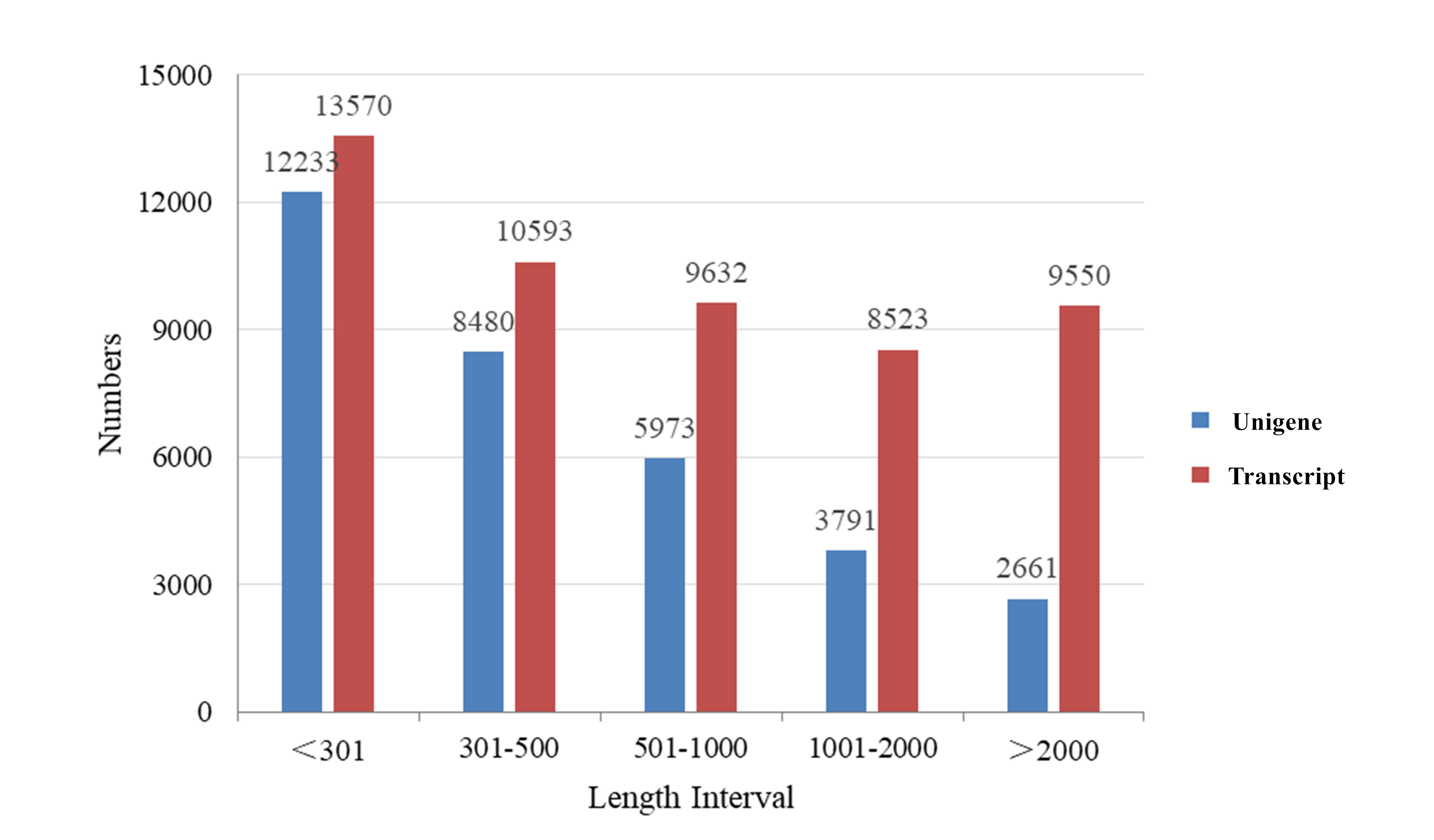

Supplement: Supplementary file 1 — Additional file 1: Figure S1. Length distribution of C. texanus unigenes and transcript. [file 13071_2019_3843_MOESM1_ESM.jpg]

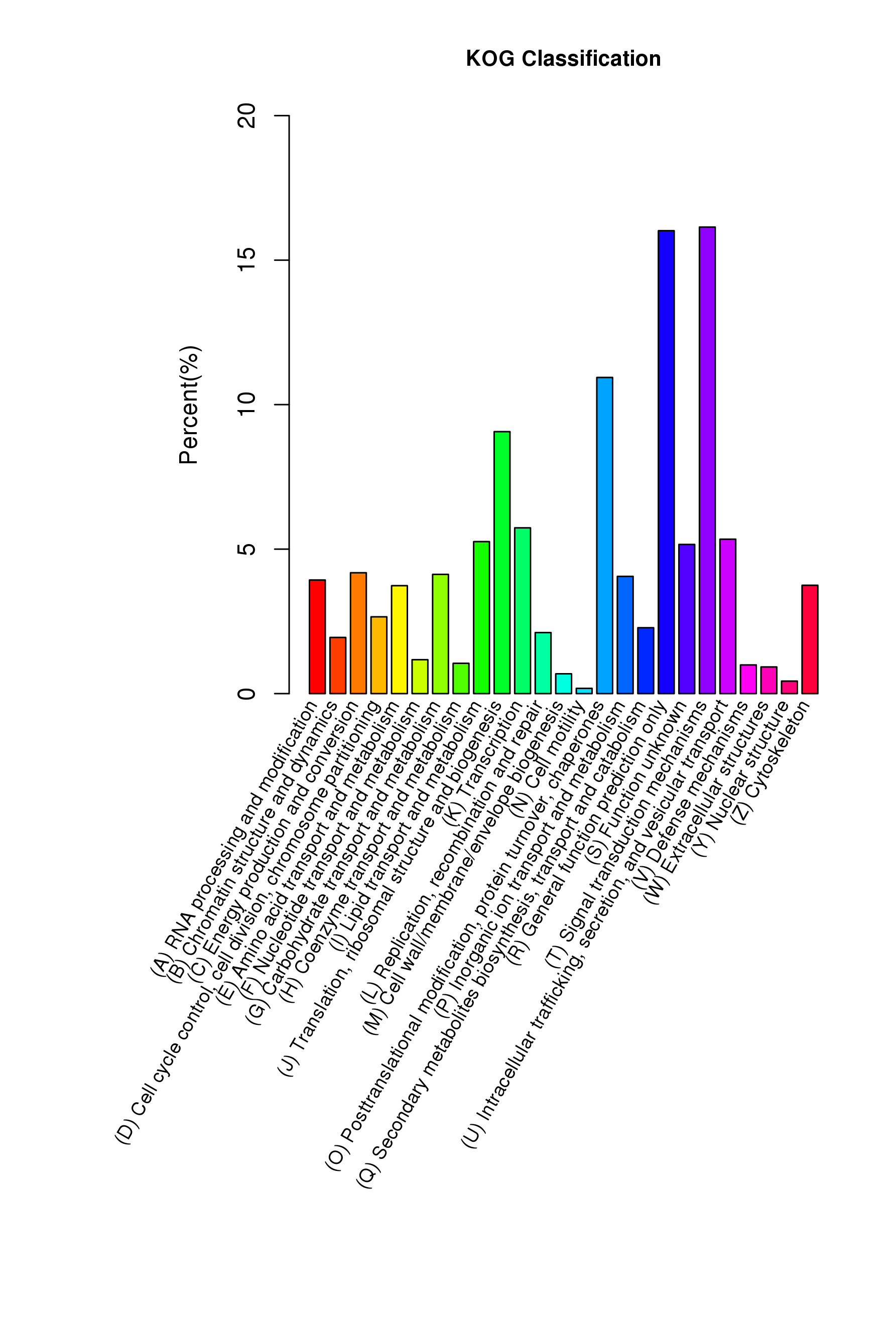

Supplement: Supplementary file 2 — Additional file 2: Figure S2. KOG classification of C. texanus unigenes. [file 13071_2019_3843_MOESM2_ESM.jpg]

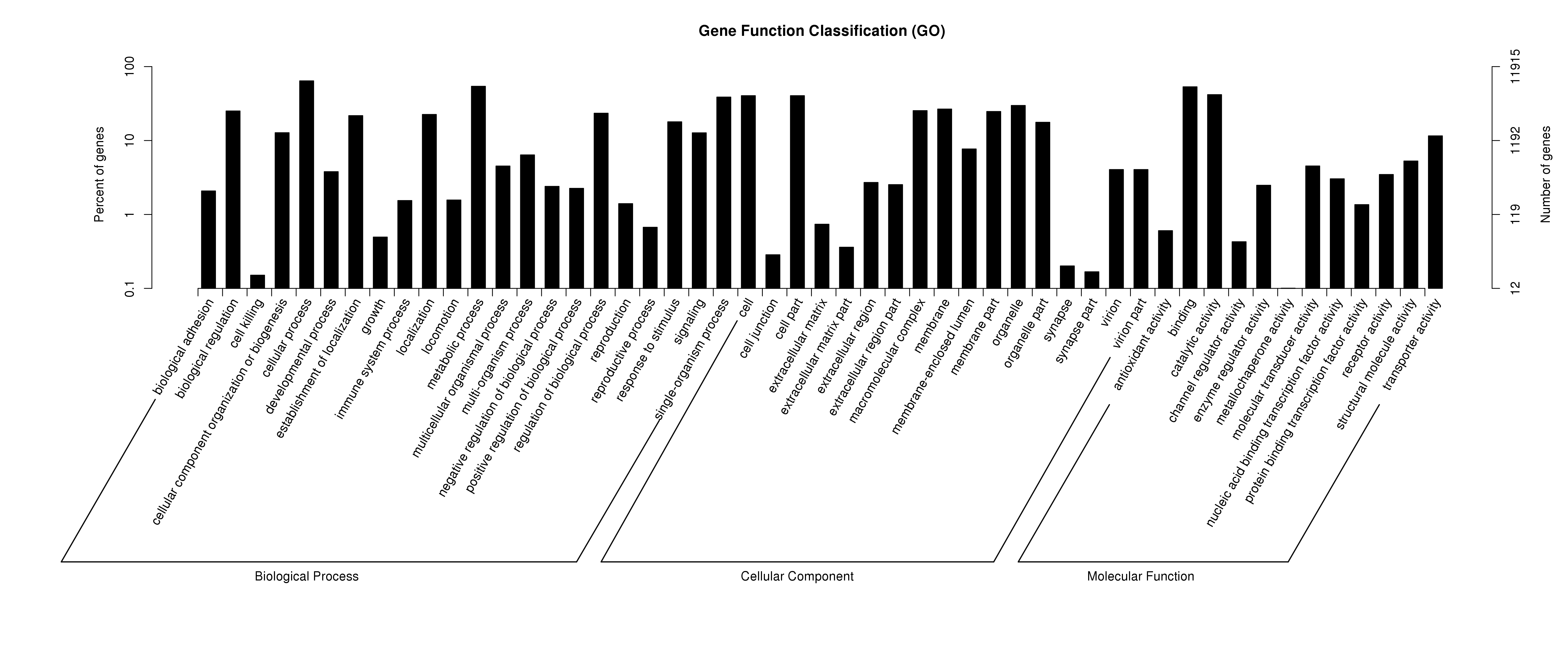

Supplement: Supplementary file 3 — Additional file 3: Figure S3. GO annotation of C. texanus unigenes. [file 13071_2019_3843_MOESM3_ESM.jpg]

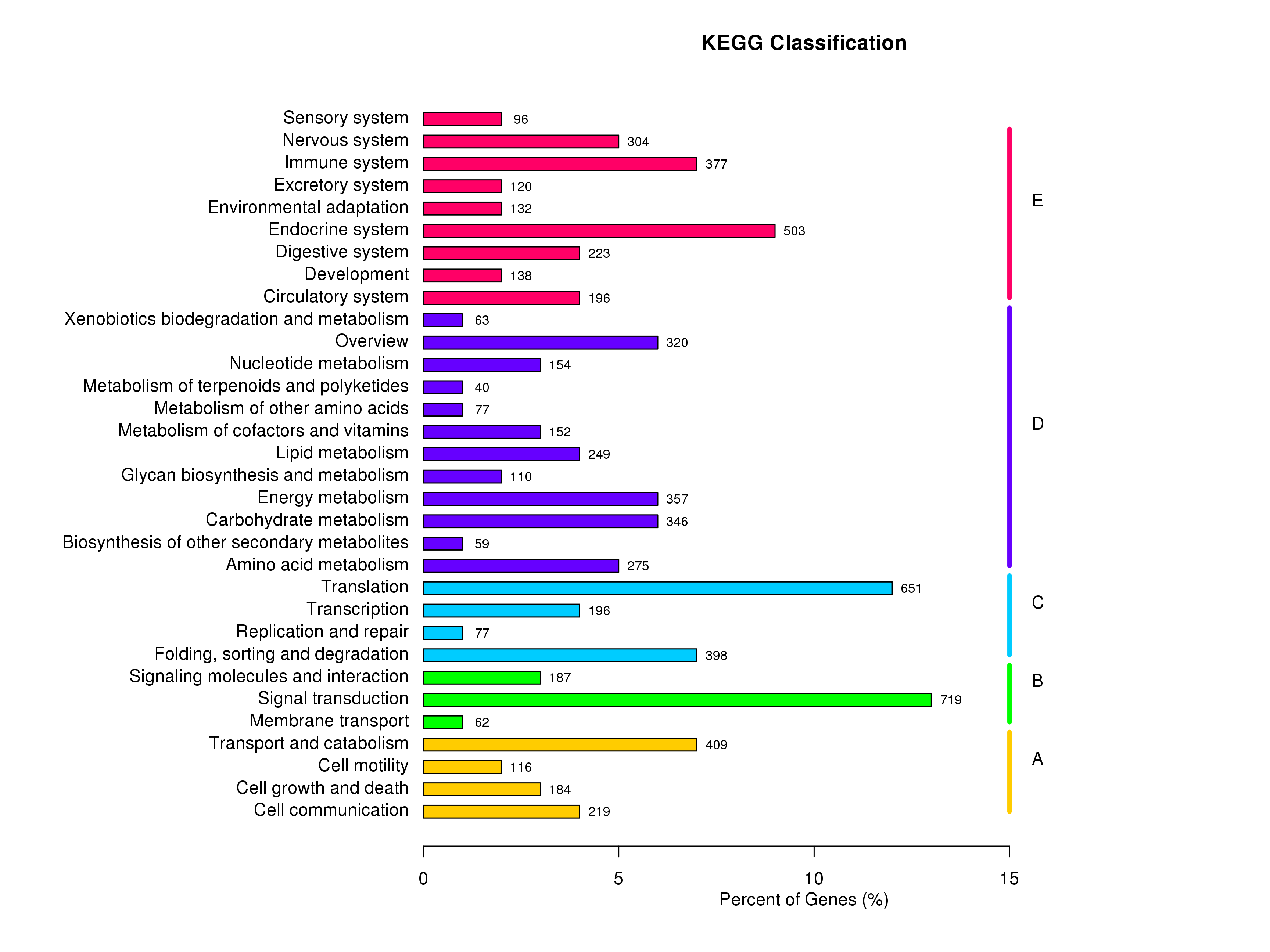

Supplement: Supplementary file 4 — Additional file 4: Figure S4. KEGG pathway analysis of C. texanus unigenes. [file 13071_2019_3843_MOESM4_ESM.jpg]

Length Distribution

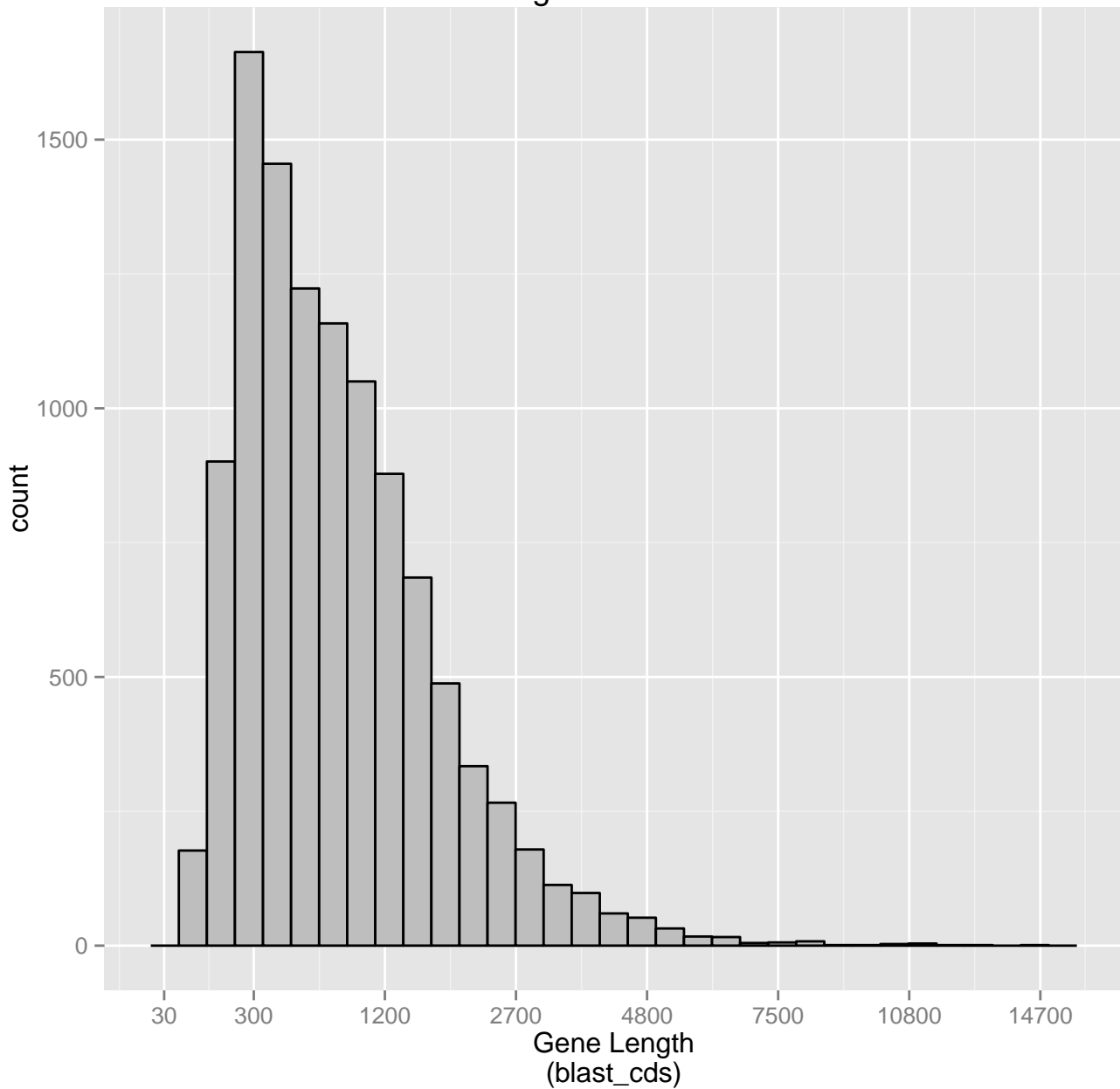

Supplement: Supplementary file 5 — Additional file 5: Figure S5. Length distribution of CDS determined by Blastx program. [file 13071_2019_3843_MOESM5_ESM.pdf]

Length Distribution

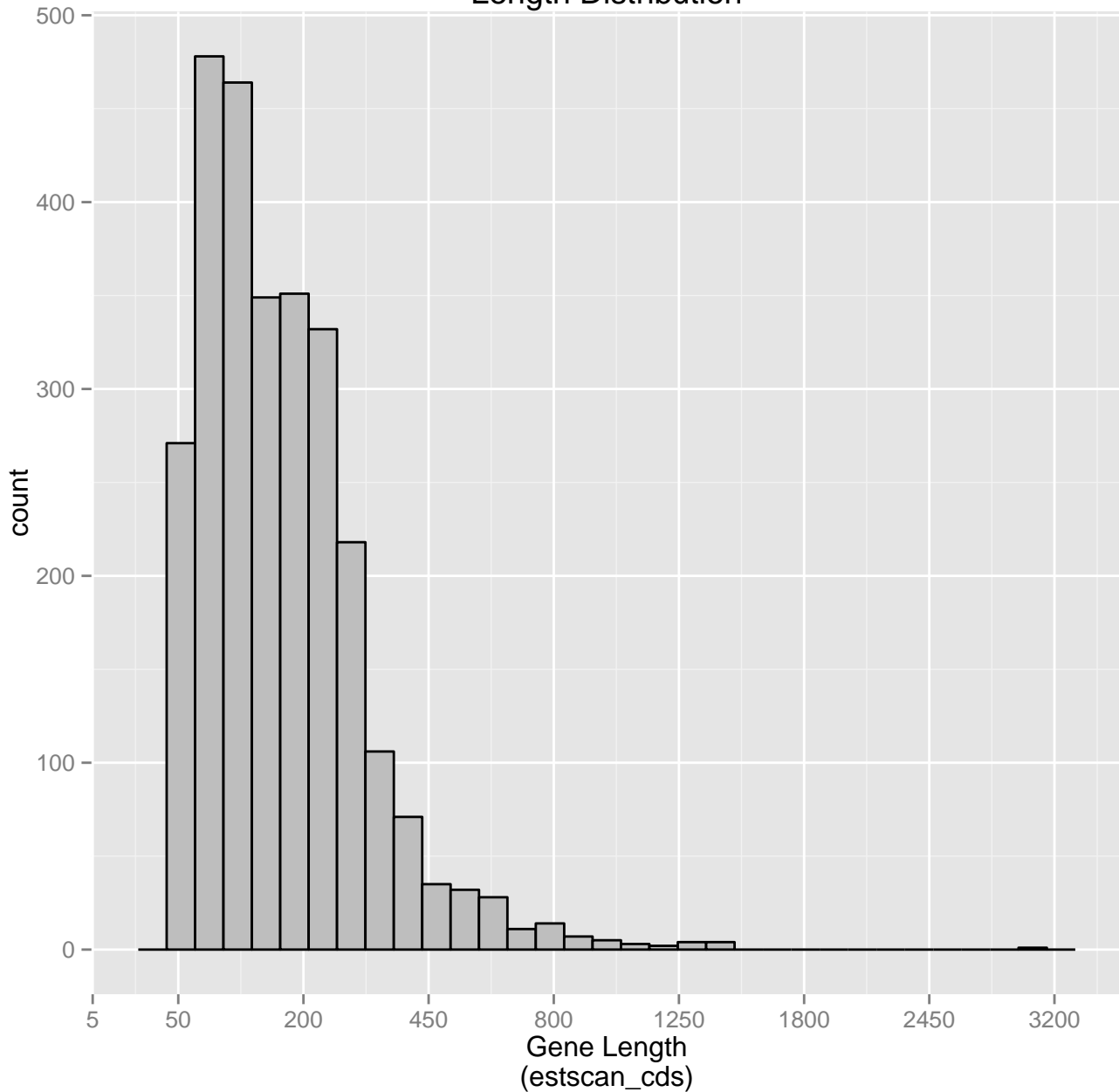

Supplement: Supplementary file 6 — Additional file 6: Figure S6. Length distribution of CDS determined by EST-Scan software. [file 13071_2019_3843_MOESM6_ESM.pdf]
